# Association of Video Game Use With Body Mass Index and Other Energy-Balance Behaviors in Children

**DOI:** 10.1001/jamapediatrics.2020.0202

**Published:** 2020-04-06

**Authors:** William Goodman, Sarah E. Jackson, Ethna McFerran, Richard Purves, Ian Redpath, Rebecca J. Beeken

**Affiliations:** 1 Research Department of Behavioural Science and Health, University College London, London, England; 2 School of Medicine Dentistry and Biomedical Sciences, Queens University Belfast, Belfast, United Kingdom; 3 Institute for Social Marketing, University of Stirling, Stirling, United Kingdom; 4 The Behaviouralist, London, England; 5 Leeds Institute of Health Sciences, University of Leeds, Leeds, England

## Abstract

**Importance:**

Childhood obesity is one of the biggest public health threats facing the UK, and video game use is considered a risk behavior for obesity among children. However, few studies have explored the prospective association between video game use and body mass index (BMI) or the potential mediators of this association.

**Objectives:**

To investigate whether a longer-term association exists between video game use at a young age and BMI SD score in later years, independent of television use, and to ascertain whether this association is mediated by other energy-balance behaviors.

**Design, Setting, and Participants:**

This cohort study is a secondary analysis of data from the Millennium Cohort Study, a nationally representative sample of children who were born in the UK between September 1, 2000, and January 31, 2002, that focused on data collected when the children were aged 5, 7, 11, and 14 years. Data for all variables, except BMI, were provided by parental or caregiver reporting if the children were younger than 14 years of age. At age 14 years, the children self-reported their own behavior. Initial data analysis was conducted between September 18, 2018, and September 28, 2018, with supplementary analyses conducted from October 7, 2019, to November 22, 2019.

**Main Outcomes and Measures:**

The main outcome variable was BMI SD scores, with video game use as the exposure variable of interest. Physical activity, bedtime regularity, sugar-sweetened beverage consumption, and high-calorie food consumption were included as potential mediating behaviors.

**Results:**

The full sample comprised 16 376 children and had a nearly equal number of boys (8393 [51.3%]) and girls (7983 [48.7%]). Every 1 SD increase in the number of hours of video game use at age 5 years was associated with a β = 0.018 higher BMI SD score at age 14 years (95% CI, 0.004-0.032). A small partial mediation of this association was found (direct association: β = 0.017 [95% CI, 0.003-0.031]; indirect association: β = 0.0011 [95% CI, 0.0003-0.0019]), suggesting that irregular bedtimes and higher consumption of sugar-sweetened beverages were mediators. The mediation model accounted for 36.7% (95% CI, 35.5-37.8) of the variance of the BMI SD score at age 14 years.

**Conclusions and Relevance:**

Results of this study suggest a small (and not clinically meaningful) association between video game use in early childhood and higher BMI in later years, which may be mediated by irregular bedtimes and higher consumption of sugar-sweetened beverages. Future interventions to prevent childhood obesity should incorporate health promotion in mainstream video games to target children most at risk because of their high level of video game use.

## Key Points

**Question:**

What are the mediating energy-balance behaviors between video game use and body mass index?

**Findings:**

In this secondary analysis of cohort data including 16 376 children in the UK, video game use at age 5 years was associated with higher body mass index SD score at age 14 years. This association was partially mediated by the consumption of sugar-sweetened beverages and the regularity of children’s bedtimes.

**Meaning:**

Findings of this study indicate that consumption of sugar-sweetened beverages and regularity of bedtimes appeared to be associated with higher body mass index among children with greater video game use early in life, but this association was small and did not seem clinically meaningful.

## Teaser

This secondary analysis examines survey data collected in the Millennium Cohort Study in the UK for variables and behaviors likely to be associated with weight gain over the long term among children aged 5 to 14 years.

## Introduction

Childhood obesity is a pressing challenge and a public health priority in the 21st century, with more than a third of children in the UK experiencing either overweight or obesity by the end of primary school.^1,2^ The central cause of obesity is the imbalance between calorie expenditure and intake, resulting from greater consumption of energy-dense foods and increased sedentary lifestyles.^3^ Video game use is considered a risk behavior for developing obesity; it increases sedentary time and may be associated with consumption of unhealthy beverages and snacks.^4^ However, few studies have looked at the association between video game use and body mass index (BMI; calculated as weight in kilograms divided by height in meters squared), independent of measures of general screen time.^5^

Studies that have investigated this association have found mixed evidence.^6,7,8,9,10^ A study of a nationally representative sample of 2831 children found a significant curvilinear association between a moderate amount of video game playing and a higher weight status, compared with very little or a high amount of video game playing.^6^ An online cross-sectional study of 562 adult participants found that male video game players reported a higher BMI than nonplayers.^7^ However, a meta-analysis of 6 cross-sectional studies (N = 1722) suggested that video game use was not associated with having body fat (*r* = 0.07; 95% CI, −0.048 to 0.188),^8^ and findings from this meta-analysis were confirmed by subsequent cross-sectional studies of adolescents.^9,10^ No study, however, has investigated the association of video game use with BMI over time, to our knowledge.

Video game use from 5 to 13 years of age is relatively stable,^11^ but sustained engagement in a sedentary behavior is likely to be associated with notable changes in weight over the long term. In line with this finding, 1 study reported that children’s screen (television and video game) time at baseline (age 5-10 years) was associated with BMI *z* scores at follow-up (age 8-13 years) (β = 0.02; 95% CI, 0.01-0.02).^12^ Therefore, cross-sectional studies may not be able to demonstrate an association between video game use and BMI in the short term, but prospective studies may capture changes that have occurred as a consequence of longer-term engagement.

In addition, video game use may be associated with other energy-balance behaviors that have been associated with obesity. Associations have been demonstrated between greater video game use and increased snacking and sugar-sweetened beverage (SSB) consumption as well as reduced physical activity and sleep duration.^8,13,14,15,16,17,18^ Meta-analyses have demonstrated that these energy-balance behaviors are associated with a higher risk of obesity.^19,20^ The role that the amount of video game use plays in poor energy-balance behaviors may explain why playing video games at an early age can be a factor in higher body weight over time.

The present study aimed to build on previous research by investigating whether a longer-term association exists between video game use at an early age and subsequent BMI, independent of television use. We also explored whether SSB consumption, physical activity, regular bedtimes, and high-calorie food consumption are mediating factors between video game use and high BMI. We hypothesized that (1) video game use would be positively associated with BMI while controlling for television use at baseline, and (2) higher levels of SSB and snack consumption, lower levels of physical activity, and less regular bedtimes would mediate this association.

## Methods

### Sample and Study Design

This cohort study is a secondary analysis of survey data collected by the Millennium Cohort Study,^21^ a nationally representative prospective birth cohort study of 19 517 children born in the UK from September 1, 2000, to January 31, 2002. The present study focused on the most recent data collected when the children were aged 5, 7, 11, and 14 years. Ethical approval before every wave of data collection in the Millennium Cohort study was obtained from the London Multi-Centre Research Ethics Committee, Yorkshire Multi-Centre Research Ethics Committee, and Yorkshire and The Humber—Leeds East and National Research Ethics Service Committee London—Central.^22^ Informed written consent for participants aged 5, 7, and 11 years was obtained from the parents or caregivers, who were asked to respond to the survey or report data for themselves and their children; for those aged 14 years, written informed consent was obtained from the children with permission from their parents.

### Measures

Data for all variables, except BMI, at ages 5, 7, and 11 years were provided by parental or caregiver reporting. At age 14 years, the children were asked to report their own behavior.

#### Video Game and Television Use

The main exposure variable was weekday video game use. The scale used 6 categories, ranging from none to more than 7 hours per weekday. After reviewing the distribution of the data, we collapsed the exposure variable into 4 categories of video game play per weekday: none, less than an hour, 1 to 3 hours, and 3 or more hours.

Television use was measured on a scale composed of 6 categories ranging from none to more than 7 hours per weekday spent watching television, videos, or DVDs. In line with previous studies, this study derived a binary variable to show high levels of television use per weekday: less than 3 hours (0) or 3 or more hours (1).^23^

#### Physical Activity and Regularity of Bedtimes

Physical activity at ages 5, 7, and 11 years was measured from parental reports of the number of days per week that their children took part in a sports club or a class that involved physical activity rated on a scale of 1 (5 or more days per week) to 6 (less than 1 day per week or not at all). As was done in previous studies, a binary measure of physical activity was derived to obtain a measure of low levels of physical activity: at least 1 day per week (0) or less often or not at all (1).^23^ Children aged 14 years were asked to estimate the number of days per week that they engaged in moderate or vigorous physical activity rated on a scale of 1 (every day) to 5 (not at all). For the present study, the categories of 1 to 2 days and not at all were condensed into the less often category.

The regularity with which children went to bed at the same time each night was measured at ages 5, 7, and 11 years based on a scale of 1 (no, never, or almost never) to 4 (yes, always). A binary variable of bedtime regularity was derived and categorized as no (0) or yes (1), in line with previous studies.^23^

#### Consumption of SSBs and High-Calorie Food

For SSB consumption at ages 5 and 7 years, a binary variable was calculated on the basis of whether the children drank SSBs in between meal times: yes (1) or no (0). The parents, when their children were aged 11 years, and the children at age 14 years were asked about the frequency of SSB consumption on a scale of 1 (once a day or more) to 3 (less often than once a week).^24^

For high-calorie food consumption at ages 5 and 7 years, binary variables were derived from whether the children ate any high-fat, high-salt, or high-sugar food (cakes, sweets, chocolate): yes (1) or no (0).^23^ At age 14 years, children were asked to indicate the frequency of their fast food consumption, on a scale of 1 (more than once a day) to 7 (never). On the basis of data distribution, fast food consumption was condensed into 4 categories, ranging from 1 (once a day or more) to 4 (less than once a month).

#### BMI SD Score and Covariates

The outcome variable for this study was the BMI SD score. At each home visit in the Millennium Cohort Study, a trained researcher measured height to the closest millimeter with a stadiometer (Leicester Height Measure; Tanita Corp) and weight to the nearest 100 g with a scale (Tanita HD-305; Tanita Corp). The BMI SD score was calculated from the UK 1990 growth reference chart assessing sex, height, weight, and age in children,^25^ using a spreadsheet (Microsoft Excel 2010, Microsoft Corp).

The covariates were sex, race/ethnicity (white or other), and socioeconomic status (SES) calculated using quintiles of family income that was adjusted according to the modified Organisation for Economic Co-operation and Development scale.^26^ Table 1 outlines the variables available at each data collection wave and the analysis type (cross-sectional or mediation) in which they were used.

**Table 1.** Summary of Variables Used in the Analyses and the Age at Which They Were Available

Variable	Age 5 y	Age 7 y	Age 11 y	Age 14 y
Covariates				
Sex	M and C	C	C	C
Race/ethnicity	M and C	C	C	C
SES	M and C	C	C	C
Independent variables/mediators				
Television use	M^a^ and C	C	C	C
Physical activity	C	M and C	M and C	C
High-calorie food consumption	C	M and C	NA	NA
Frequency of fast food consumption	NA	NA	NA	C
SSB consumption	C	M and C	NA	NA
Frequency of SSB consumption	NA	NA	M and C	C
Regular bedtime	C	M and C	M and C	NA
Video game use	M and C	C	C	C
Outcome variable				
BMI-SDS	M^a^ and C	C	C	M and C

Abbreviations: BMI-SDS, body mass index SD score; C, included in cross-sectional analysis; M, included in mediation analysis; NA, not applicable; SES, socioeconomic status; SSB, sugar-sweetened beverage.

^a^ Was used as a covariate in the mediation analysis.

### Statistical Analysis

Descriptive statistics were stratified by video game use. Missing value analysis found that 17% of 16 376 values were missing and 46% of 524 032 cases had at least 1 piece of missing data, and the Little *t* test ascertained that the data were not missing completely at random. Multiple imputation was conducted to account for the missing data, with all the variables used in the analyses included. Missing data from variables considered to be stable over time, such as sex and race/ethnicity, were imputed whenever possible from all time points.^27^ Body Mass Index SD scores less than –5 or greater than 5 were removed because they were deemed to be extreme values.^28^ This removal provided a sample of 16 376 participants for the analyses.

For all analyses, CIs were used to judge statistical significance, except for the development of the mediation model in which *P* < .20 was used to identify associated variables. Statistical analyses were conducted in IBM SPSS Statistics, version 24.0 (IBM), and SmartPLS, version 3.0 (SmartPLS GmbH). Initial data analysis was conducted between September 18, 2018, and September 28, 2018, with supplementary analyses conducted from October 7, 2019, to November 22, 2019.

#### Cross-Sectional Analysis

Hierarchical linear regressions were conducted at each data collection wave to analyze the associations between the variables of interest and the BMI SD score while controlling for sex, race/ethnicity, and SES. Covariates were added into the regression at the first level and followed by the energy-balance behaviors at the next level, as these variables’ association with BMI SD score had robust support in the literature. Video game use was added into the final level, as it was the variable of interest in the study and had less support in previous literature.

#### Mediation Analysis 

Path analysis was used to explore whether energy-balance behaviors mediated the association between video game use and BMI SD score. The Baron and Kenny^29^ method was initially followed to establish which variables to include in the full mediation analysis. We ran linear and logistic regressions to ascertain which variables were associated with BMI SD score and whether video game use was associated with the energy-balance mediators. These variables were controlled for sex, race/ethnicity, SES, television use, and BMI SD score at baseline (age 5 years). A post hoc power and sample size calculation was conducted to ensure enough degrees of freedom to enable the identification of the model and enough observations to enable the accurate estimation of the paths. Based on the Streiner study,^30^ the exploratory model we used could estimate a maximum of 66 parameters and the final model 45 parameters. The exploratory model had 60 parameters and the final model 44 parameters, which meant that our models were slightly overidentified. Klein^31^ claimed that between 10 and 20 observations were needed per parameter. With the exploratory model run for the present study requiring a sample size of 1200, we confirmed that the sample was adequate.

The exploratory model was refined, by removing the weakest mediators, to improve the model fit. The model fit was assessed with the comparative fit index (CFI), the Tucker-Lewis index (TLI) being greater than 0.95, and the root mean square error of approximation (RMSEA) being less than 0.05. The fitting algorithm used was partial least squares. A sensitivity analysis was also conducted in which the outcome variable (BMI SD score at age 14 years) was replaced with the change in BMI SD score from age 5 to 14 years. A further sensitivity analysis was conducted in which the independent variables were not collapsed.

## Results

### Descriptive Statistics

A total of 15 246 children participated in the data collection at age 5 years, 13 857 at age 7 years, 13 287 at age 11 years, and 11 714 at age 14 years. The full sample comprised 16 376 children and had nearly an equal number of boys (8393 [51.3%]) and girls (7983 [48.7%]), with the predominant race/ethnicity of white (ranging from 8961 of 11714 [76.5%] to 11597 of 13857 [83.7%] reported at each data collection wave). A roughly equal number of participants from each SES was also observed, with the difference ranging from 239 of 13857 (1.7%) to 704 of 11714 (6.0%) at each wave. Descriptive statistics are reported in Table 2.

**Table 2.** Descriptive Statistics for the Variables Included in the Study

Variable	Video Game Use, No. (%)																			
	Age 5 y (n = 15 246)					Age 7 y (n = 13 857)					Age 11 y (n = 13 287)					Age 14 y (n = 11 714)				
	None	<1 h	1-3 h	≥3 h	Total	None	<1 h	1-3 h	≥3 h	Total	None	<1 h	1-3 h	≥3 h	Total	None	<1 h	1-3 h	≥3 h	Total
Sex																				
Male	2177 (44.4)	3317 (50.0)	1965 (62.1)	298 (64.8)	7798 (51.1)	740 (43.1)	3225 (45.1)	2629 (60.6)	399 (69.0)	7029 (50.7)	584 (29.8)	2061 (40.3)	3394 (64.5)	583 (73.9)	6712 (50.5)	321 (15.1)	832 (28.9)	2100 (63.9)	2418 (78.6)	5877 (50.2)
Female	2730 (55.6)	3320 (50.0)	1200 (37.9)	162 (35.2)	7448 (48.9)	975 (56.9)	3919 (54.9)	1709 (39.4)	179 (31.0)	6828 (49.3)	1378 (70.2)	3049 (59.7)	1867 (35.5)	206 (26.1)	6575 (49.5)	1806 (84.9)	2042 (71.1)	1187 (36.1)	658 (21.4)	5837 (49.8)
Race/ethnicity																				
White	4134 (84.2)	5641 (85.0)	2532 (80.0)	350 (76.1)	12 705 (83.3)	1258 (73.4)	6138 (85.9)	3652 (84.2)	489 (84.6)	11 597 (83.7)	1506 (76.8)	4314 (84.4)	4389 (83.4)	659 (83.5)	10 992 (82.7)	1629 (77.3)	2238 (78.5)	2501 (76.8)	2585 (85.0)	8961 (79.5)
Other	773 (15.8)	994 (15.0)	633 (20.0)	110 (23.9)	2539 (16.7)	457 (26.6)	1005 (14.1)	686 (15.8)	89 (15.4)	2259 (16.3)	445 (23.2)	795 (15.6)	871 (16.6)	130 (16.5)	2292 (17.3)	478 (22.7)	613 (21.5)	756 (23.2)	456 (15.0)	2304 (20.5)
SES, quintile																				
Lowest	1202 (24.5)	1160 (17.5)	846 (26.8)	164 (35.9)	3375 (22.3)	540 (31.5)	1170 (16.4)	956 (22.0)	177 (30.6)	2856 (20.6)	417 (21.3)	816 (16.0)	1048 (19.9)	207 (26.2)	2530 (19.0)	300 (14.1)	432 (15.0)	630 (19.2)	550 (17.9)	2010 (17.2)
Second	1015 (20.7)	1316 (19.9)	809 (25.6)	124 (27.1)	3264 (21.6)	406 (23.7)	1357 (19.0)	962 (22.2)	120 (20.8)	2862 (20.7)	429 (21.9)	864 (16.9)	1042 (19.8)	179 (22.7)	2551 (19.2)	321 (15.1)	460 (16.0)	540 (16.4)	580 (18.9)	1983 (16.9)
Third	912 (18.6)	1358 (20.5)	593 (18.8)	76 (16.6)	2939 (19.4)	263 (15.3)	1432 (20.1)	959 (22.1)	122 (21.1)	2797 (20.2)	395 (20.1)	1053 (20.6)	1165 (22.1)	172 (21.8)	2828 (21.3)	407 (19.1)	561 (19.5)	660 (20.1)	691 (22.5)	2382 (20.3)
Fourth	872 (17.8)	1437 (21.7)	535 (17.0)	60 (13.1)	2906 (19.2)	259 (15.1)	1549 (21.7)	783 (18.1)	94 (16.3)	2699 (19.5)	365 (18.6)	1205 (23.6)	1094 (20.8)	125 (15.8)	2815 (21.2)	517 (24.3)	681 (23.7)	728 (22.1)	704 (22.9)	2687 (22.9)
Highest	897 (18.3)	1349 (20.4)	371 (11.8)	33 (7.2)	2651 (17.5)	247 (14.4)	1629 (22.8)	676 (15.6)	65 (11.2)	2623 (19.0)	356 (18.1)	1172 (22.9)	912 (17.3)	106 (13.4)	2563 (19.3)	582 (27.4)	740 (25.7)	729 (22.2)	551 (17.9)	2652 (22.6)
Television use, h																				
<3	4311 (87.9)	5923 (89.2)	2426 (76.7)	194 (42.2)	12 856 (84.8)	1475 (86.1)	6440 (90.1)	3405 (78.5)	255 (44.1)	11 582 (84.0)	1736 (88.5)	4593 (89.9)	4406 (83.8)	401 (50.8)	11 139 (84.9)	1378 (64.8)	1853 (64.5)	1957 (59.6)	1303 (42.4)	6497 (57.2)
≥3	595 (12.1)	712 (10.7)	738 (23.3)	266 (57.8)	2313 (15.2)	239 (13.9)	704 (9.9)	931 (21.5)	322 (55.8)	2198 (16.0)	226 (11.5)	517 (10.1)	853 (16.2)	388 (49.2)	1985 (15.1)	747 (35.2)	1020 (35.5)	1329 (40.4)	1772 (57.6)	4869 (42.8)
Frequency of physical activity																				
≥1 d/wk	2437 (49.7)	3690 (55.6)	1448 (45.8)	164 (35.7)	7741 (51.0)	972 (56.7)	5146 (72.0)	2785 (64.2)	337 (58.3)	9247 (67.1)	1429 (72.8)	3957 (77.4)	3857 (73.3)	491 (62.2)	9737 (74.2)	NA	NA	NA	NA	NA
<Once/ wk	2470 (50.3)	2947 (44.4)	1717 (54.2)	296 (64.3)	7433 (49.0)	743 (43.3)	1998 (28.0)	1553 (35.8)	241 (41.7)	4540 (32.9)	533 (27.2)	1153 (22.6)	1404 (26.7)	298 (37.8)	3390 (25.8)	NA	NA	NA	NA	NA
Every day	NA	NA	NA	NA	NA	NA	NA	NA	NA	NA	NA	NA	NA	NA	NA	335 (15.8)	524 (18.2)	705 (21.5)	525 (17.1)	2093 (18.4)
5-6 d/wk	NA	NA	NA	NA	NA	NA	NA	NA	NA	NA	NA	NA	NA	NA	NA	408 (19.2)	552 (19.2)	679 (20.7)	559 (18.2)	2199 (19.4)
3-4 d/wk	NA	NA	NA	NA	NA	NA	NA	NA	NA	NA	NA	NA	NA	NA	NA	697 (32.9)	991 (34.5)	1123 (34.2)	1017 (33.1)	3829 (33.7)
Less often	NA	NA	NA	NA	NA	NA	NA	NA	NA	NA	NA	NA	NA	NA	NA	681 (32.1)	806 (28.1)	776 (23.6)	971 (31.6)	3236 (28.5)
Regular bedtime																				
No	218 (4.4)	287 (4.3)	221 (7.0)	41 (8.9)	767 (5.1)	76 (4.4)	218 (3.1)	192 (4.4)	47 (8.1)	533 (3.9)	62 (3.2)	144 (2.8)	197 (3.7)	54 (6.8)	457 (3.5)	NA	NA	NA	NA	NA
Yes	4689 (95.6)	6350 (95.7)	2944 (93.0)	419 (91.1)	14 407 (94.9)	1639 (95.6)	6925 (96.9)	4146 (95.6)	531 (91.9)	13 250 (96.1)	1900 (96.8)	4966 (97.2)	5063 (96.3)	735 (93.2)	12 669 (96.5)	NA	NA	NA	NA	NA
High-calorie food consumption																				
None	1043 (21.2)	1241 (18.7)	485 (15.3)	75 (16.3)	2844 (18.8)	385 (22.4)	1429 20.0()	723 (16.7)	86 (14.9)	2626 (19.1)	NA	NA	NA	NA	NA	NA	NA	NA	NA	NA
Yes	3861 (78.7)	5392 (81.2)	2678 (84.6)	384 (83.5)	12 320 (81.2)	1330 (77.6)	5713 (80.0)	3614 (83.3)	491 (85.1)	11 154 (80.9)	NA	NA	NA	NA	NA	NA	NA	NA	NA	NA
SSB consumption																				
None	3489 (71.1)	4740 (71.5)	2136 (67.6)	305 (66.4)	10 674 (70.4)	1268 (73.9)	5260 (73.7)	3025 (69.7)	367 (63.5)	9927 (72.0)	NA	NA	NA	NA	NA	NA	NA	NA	NA	NA
Yes	1415 (28.9)	1894 (28.5)	1026 (32.4)	154 (33.6)	4490 (29.6)	447 (26.1)	1880 (26.3)	1312 (30.3)	211 (36.5)	3852 (28.0)	NA	NA	NA	NA	NA	NA	NA	NA	NA	NA
Frequency of SSB consumption																				
≥Once/d	NA	NA	NA	NA	NA	NA	NA	NA	NA	NA	549 (28.0)	1483 (29.0)	1778 (33.8)	281 (35.6)	4094 (31.2)	389 (18.3)	578 (20.2)	768 (23.5)	947 (31.0)	2684 (23.8)
1-6 d/wk	NA	NA	NA	NA	NA	NA	NA	NA	NA	NA	561 (28.6)	1477 (28.9)	1589 (30.2)	252 (31.9)	3879 (29.6)	821 (38.8)	1223 (42.8)	1586 (48.5)	1422 (46.6)	5057 (44.8)
Less often	NA	NA	NA	NA	NA	NA	NA	NA	NA	NA	851 (43.4)	2145 (42.0)	1892 (36.0)	256 (32.4)	5146 (39.2)	904 (42.8)	1059 (37.0)	914 (28.0)	682 (22.4)	3559 (31.5)
Frequency of fast food consumption																				
≥Once/d	NA	NA	NA	NA	NA	NA	NA	NA	NA	NA	NA	NA	NA	NA	NA	37 (1.7)	45 (1.6)	68 (2.1)	62 (2.0)	212 (1.9)
1-6 d/wk	NA	NA	NA	NA	NA	NA	NA	NA	NA	NA	NA	NA	NA	NA	NA	459 (21.7)	703 (24.5)	910 (27.8)	965 (31.6)	3041 (26.9)
At least once/mo	NA	NA	NA	NA	NA	NA	NA	NA	NA	NA	NA	NA	NA	NA	NA	888 (41.9)	1275 (44.5)	1461 (44.7)	1343 (43.9)	4967 (43.9)
Less often	NA	NA	NA	NA	NA	NA	NA	NA	NA	NA	NA	NA	NA	NA	NA	735 (34.7)	842 (29.4)	832 (25.4)	686 (22.4)	3098 (27.4)
BMI-SDS, mean (SD)	0.4 (1.1)	0.4 (1.1)	0.5 (1.1)	0.5 (1.2)	0.4 (1.1)	0.4 (1.2)	0.4 (1.1)	0.5 (1.2)	0.5 (1.2)	0.4 (1.2)	0.6 (1.2)	0.6 (1.2)	0.7 (1.2)	0.8 (1.3)	0.7 (1.2)	0.7 (1.1)	0.7 (1.2)	0.7 (1.2)	0.7 (1.3)	0.7 (1.2)

Abbreviations: BMI-SDS, body mass index SD score; NA, not applicable; SES, socioeconomic status; SSB, sugar-sweetened beverage.

### Cross-Sectional Analysis

Associations between the covariates and BMI SD scores were consistent across all ages (sex: ranging from β = −0.05 [95% CI, −0.09 to −0.02] at age 5 years to β = 0.06 [95% CI, 0.01-0.12] at age 14 years; race/ethnicity: ranging from β = −0.30 [95% CI, −0.35 to −0.25] at age 5 years to β = −0.10 [95% CI, −0.18 to −0.03] at age 14 years; SES: ranging from β = −0.02 [95% CI, −0.03 to −0.01] at age 5 years to β = −0.09 [95% CI, −0.11 to −0.07] at age 14 years). Associations between energy-balance variables and BMI SD scores were less consistent across ages (physical activity, ≥1 d/wk vs <1 d/wk: ranging from β = −0.02 [95% CI, −0.06 to 0.02] at age 5 years to β = 0.04 [95% CI, −0.02 to 0.10] at age 11 years; SSB consumption: ranging from β = ≤0.01 [95% CI, −0.04 to 0.04] at age 5 years to β = 0.01 [95% CI, −0.03 to 0.05] at age 7 years; high-calorie food consumption: ranging from β = −0.03 [95% CI, −0.07 to 0.02] at age 5 years to β = −0.03 [95% CI, −0.09 to 0.03] at age 7 years; regular bedtimes: ranging from β = 0.14 [95% CI, 0.06-0.22] at age 5 years to β = 0.06 [95% CI, −0.05 to 0.17] at age 11 years). Video game use, however, was associated with the BMI SD score at ages 5 years (β = 0.03; 95% CI, 0.01-0.05) and 7 years (β = 0.05; 95% CI, 0.02-0.07). Results of the fully adjusted regressions are shown in Table 3.

**Table 3.** Fully Adjusted Linear Regression Models Conducted at Each Wave for Associations With Body Mass Index SD Score

Variable	Difference (95% CI)^a^			
	Millennium Cohort Study waves (n = 16 376)			
	Age 5 y	Age 7 y	Age 11 y	Age 14 y
Sex				
Male vs female	−0.05 (−0.09 to −0.02)	−0.06 (−0.09 to −0.02)	−0.13 (−0.17 to −0.08)	0.06 (0.01 to 0.12)
Race/ethnicity				
White vs other	−0.30 (−0.35 to −0.25)	−0.16 (−0.21 to −0.11)	−0.06 (−0.11 to <0.01)	−0.10 (−0.18 to −0.03)
SES, income quintiles,				
Lowest to highest	−0.02 (−0.03 to −0.01)	−0.03 (−0.04 to −0.01)	−0.05 (−0.07 to −0.04)	−0.09 (−0.11 to −0.07)
Television use, h				
None to <3 h vs ≥3 h	0.09 (0.04 to 0.14)	0.07 (0.01 to 0.12)	0.15 (0.09 to 0.20)	0.11 (0.05 to 0.16)
Frequency of physical activity				
At least 1 d/wk vs <1 d/wk	−0.02 (−0.06 to 0.02)	−0.02 (−0.10 to 0.07)	0.04 (−0.02 to 0.10)	NA
Every day, 5-6 d/wk, 3-4 d/wk, less often	NA	NA	NA	0.09 (0.07 to 0.12)
SSB				
No consumption vs consumption	<0.01 (−0.04 to 0.04)	0.01 (−0.03 to 0.05)	NA	NA
Frequency of SSB consumption				
≥Once/d, 1-6 d/wk, less often	NA	NA	−0.01 (−0.04 to 0.01)	−0.03 (−0.06 to <0.01)
High-calorie food				
No consumption vs consumption	−0.03 (−0.07 to 0.02)	−0.03 (−0.09 to 0.03)	NA	NA
Frequency of fast food consumption				
≥Once/d, 1-6 d/wk, once/mo, less often	NA	NA	NA	0.09 (0.06 to 0.11)
Bedtimes				
Regular bedtime vs irregular bedtime	0.14 (0.06 to 0.22)	0.11 (0.01 to 0.21)	0.06 (−0.05 to 0.17)	NA
Video game use, h				
None, <1 h, 1-3 h, ≥3 h	0.03 (0.01 to 0.05)	0.05 (0.02 to 0.07)	<0.01 (−0.04 to 0.04)	0.01 (−0.01 to 0.03)

Abbreviations: NA, not applicable; SES, socioeconomic status; SSB, sugar-sweetened beverage.

^a^ Difference refers to the standardized β regression coefficients.

### Mediation Analysis

Regular bedtime at age 7 years (odds ratio [OR], 1.20 [95% CI, 1.05-1.37]; β = 0.142 [95% CI, 0.051-0.232]), SSB consumption at age 7 years (OR, 1.08 [95% CI, 1.03-1.13]; β = 0.037 [95% CI, 0.001-0.073]) and 11 years (β = −0.043 [95% CI, −0.062 to −0.024]; β = −0.023 [95% CI, −0.046 to −0.001]), physical activity at age 7 years (OR, 1.03 [95% CI, 0.99-1.08]; β = 0.045 [95% CI, 0.006-0.084]), and high-calorie food consumption at age 7 years (OR, 1.10 [95% CI, 1.04-1.16]; β = 0.037 [95% CI, −0.016 to 0.090]) were associated (*P* < .20) with video game use at age 5 years and the BMI SD score at age 14 years. Therefore, because all energy-balance behavior measures were associated at age 7 years, these measures were used in the exploratory mediation models, with the exception of SSB consumption at age 11 years because of the high multicollinearity with SSB consumption at age 7 years. Of the 2 measures, SSB consumption at age 7 years was retained to be consistent with the age at assessment of the other mediating behaviors in the model. The results of the exploratory regressions for the mediation analysis are outlined in Table 4. The exploratory models were subsequently pruned, with nonsignificant mediation variables removed to improve the model fit (eFigures 1 and 2 in the Supplement).

**Table 4.** Logistic and Linear Regressions for Associations With Body Mass Index SD Score and Energy-Balance Behaviors

Variable	Hours of video game use at age 5 y, OR (95% CI)	BMI-SDS at age 14 y, difference (95% CI)^a^
Physical activity, age 7 y		
At least 1 d/wk vs <1 d/wk	1.03 (0.99 to 1.08)	0.045 (0.006 to 0.084)
Physical activity, age 11 y		
At least 1 d/wk vs <1 d/wk	1.04 (0.99 to 1.09)	0.030 (−0.027 to 0.088)
High-calorie food consumption, age 7 y		
No consumption vs consumption	1.10 (1.04 to 1.16)	0.037 (−0.016 to 0.090)
Bedtimes, age 7 y		
Regular bedtime vs irregular bedtime	1.20 (1.05 to 1.37)	0.142 (0.051 to 0.232)
Bedtimes, age 11 y		
Regular bedtime vs irregular bedtime	1.06 (0.95 to 1.18)	0.143 (−0.021 to 0.308)
Video game use, age 5 y		
None, <1 h, 1-3 h, ≥3 h	NA	0.027 (0.004 to 0.050)
SSB consumption, age 7 y		
No consumption vs consumption	1.08 (1.03 to 1.13)	0.037 (0.001 to 0.073)
SSB consumption, age 11 y		
≥Once/d, 1-6 d/wk, less often	−0.043 (−0.062 to −0.024)^b^	−0.023 (−0.046 to −0.001)^b^

Abbreviations: BMI-SDS, body mass index SD score; NA, not applicable; OR, odds ratio; SSB, sugar-sweetened beverage.

^a^ Difference refers to the standardized β regression coefficients.

^b^ Reflects the difference (95% CI), not the OR.

The final model included regular bedtime and SSB consumption at age 7 years. The results of the model fit measures indicated a good fit of the model (CFI = 0.999; TLI = 0.974; RMSEA = 0.021). The standardized bias-corrected bootstrapping results showed that video game use at 5 years was associated with regular bedtimes (β = 0.031; 95% CI, 0.013-0.049) and SSB consumption (β = 0.029; 95% CI, 0.013-0.045) at age 7 years, and both regular bedtimes (β = 0.022; 95% CI, 0.008-0.036) and consumption of SSB (β = 0.013; 95% CI, 0.001-0.025) at age 7 years were associated with the BMI SD score at age 14 years (Figure). The total association (direct and indirect associations) of video game use with BMI SD score was statistically significant (β = 0.018; 95% CI, 0.004-0.032), and the direct association of video game use with BMI SD score was also statistically significant (β = 0.017; 95% CI, 0.003-0.031), indicating partial mediation. The total indirect association was statistically significant (β = 0.0011; 95% CI, 0.0003-0.0019), which suggests that the combination of the variables successfully mediated a small proportion of the association between video game use and BMI SD score. The specific indirect pathways for regular bedtimes (β = 0.0005; 95% CI, 0.0001-0.0011) and SSB consumption (β = 0.0004; 95% CI, 0.0001-0.0008) were both statistically significant. The final mediation model accounted for 36.7% (95% CI, 35.5-37.8) of the variance of the BMI SD score at age 14 years.

**Figure.**
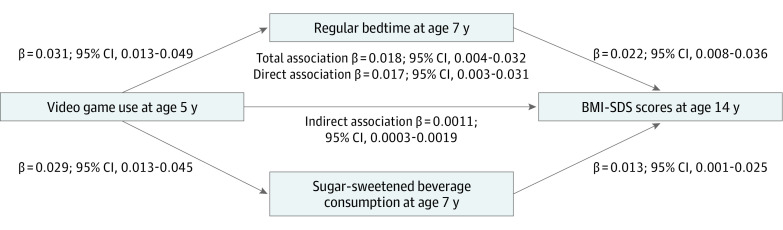
Final Mediated Pathway From Video Game Use to the Body Mass Index SD Score Standardized regression coefficients are shown with the arrows pointing to the outcome variable.

The sensitivity analyses with change in BMI SD score as the outcome and the independent variables that were not collapsed had similar results (eFigures 3 and 4 in the Supplement).

## Discussion

To our knowledge, this study was the first to investigate the prospective association between video game use and BMI SD score while controlling for television use. Greater video game use at age 5 years was associated with a higher BMI SD score at age 14 years, although this association was small. This association appeared to be partially mediated by the regularity of bedtimes and SSB consumption, athough indirect associations were small. These mediating pathways are consistent with previous research that reported increased video game use was associated with less regular bedtimes^20,32^ and greater consumption of SSBs^19^ and that irregular bedtimes and higher SSB consumption were associated with elevated BMI.^13,15,33,34,35^ However, we believe that the present study was the first to explicitly test this mediation model.

A potential explanation for why SSB consumption was found as a mediator of the association between video game use and BMI is that many video games have embedded in-game advertisements, which often promote SSBs. These advertisements take different forms, such as posters and billboards displayed in the environment and in-game consumption of branded SSBs.^36^ Brands invest a lot of advertising money into video games and video gaming events; Red Bull, for instance, has a strong presence in the gaming community.^37^ Research has shown that in-game advertisements can make a strong impression on implicit memory, which could have a subconscious implication for later actions.^38^ This process can be explained by the theory of human associative memory, which posits that in-game presentation of a brand and repeated exposure to this presentation can link the brand with the game, which over time can affect brand image.^39^ Furthermore, the established association between SSB consumption and obesity^13,15^ may help to explain the path from video game use to BMI SD score through the consumption of SSBs.

An explanation for why irregular bedtimes were found to mediate the association between video game use at age 5 years and the BMI SD score at age 14 years is that video game use before bedtime could be a factor in shorter sleep duration,^18^ which previous research has found to be associated with increased calorie intake and the development of obesity.^33,35^ However, although overall the mediation model accounted for a substantial amount of the variance of the BMI SD score at age 14 years (36.7%), the effect sizes for the mediators in this study, despite being statistically significant, were small; thus, further research is needed to confirm these associations.

Although the prospective association between video game use and BMI SD score was consistent with previous research that explored the association between screen time and BMI SD score,^12^ findings from other research have suggested that a 0.25 change in BMI SD score was needed for it to be a cardiovascular risk factor in children.^40^ Therefore, these associations are unlikely to be clinically meaningful. The cross-sectional regressions also indicated that video game use may be differentially associated with BMI SD scores at different ages, with stronger associations observed at the younger ages (5 and 7 years) vs the older ages. This finding may reflect the increasing role that other behaviors play as a child gets older, weakening the association between this initial behavior and BMI. The association between other variables and BMI SD scores also appeared to differ over time. For example, high-calorie food consumption was associated with BMI at age 14 years but not at the younger ages. This finding could reflect children’s growing independence from their parents, who had more control over their food intake at a younger age. However, the associations were small at all ages.

The results of this study could have implications for interventions that seek to target children’s health behavior through video games. Previous research has found that parental restriction of screen time, specifically television use, can be effective in controlling children’s behavior.^41^ Therefore, future research should investigate whether the principle behind these interventions could be applied to video game use to increase sleep regularity and duration among children. Because of the immersive nature of video games, players often remain engaged for prolonged periods and thus are exposed to more in-game advertisements.^42^ The implications of in-game SSB advertising and whether the restriction of gameplay time could alleviate the consumption of SSBs need to be studied. Furthermore, informing parents of the possible implications of video game use for their children may help raise awareness of the negative association between video game use and BMI. Nonetheless, these results may guide future interventions to prevent weight gain in childhood by encouraging health promotion in mainstream video games and helping to identify children most at risk because of their high levels of video game use.

### Limitations

This study has several limitations. First, the mediation analysis we conducted could not establish causality, so care must be taken when interpreting the study findings. Further experimental research is needed to investigate the pathway from video game use to BMI through SSB consumption and regular bedtimes to confirm these associations. Second, most of the variables were either parent- or self-reported, which could have introduced social desirability bias in the responses.^43^ Because this study relied on secondary data, the best measures could not be selected for our research question. Previous research into the associations between snacking and BMI SD score recorded snacking frequency and percentage of overall caloric intake, which are more representative of behavior than the type of snacks the children ate.^44,45^ In addition, these behaviors have been found to be associated with BMI in short-term studies; therefore, assessing these behaviors over the course of a number of years may not reflect current realities.

## Conclusions

Results of this secondary analysis indicated that early exposure to video games was associated with higher BMI SD score in later years, which may be partially explained by the irregularity of bedtimes and high consumption of SSBs among children who played more video games. However, a causal relationship could not be established by the mediation model used in the study. Further research is needed to identify whether a clinically meaningful association exists between these energy-balance behaviors and BMI given that the effect sizes were small, although a substantial amount of variance of the BMI SD score at age 14 years was accounted for in the model. Future interventions should also consider health promotion in mainstream video games to target children who are most at risk for obesity.

## Notes

Supplement. **eFigure 1.** Exploratory Mediation Model (All Energy Balance Behaviours at Age 7) **eFigure 2.** Exploratory Mediation Model (Energy Balance Behaviours at Age 7 Minus Physical Activity) **eFigure 3.** Sensitivity Analysis Mediation Model, BMI-SDS Change From Age 5 to Age 14 as Outcome **eFigure 4.** Sensitivity Analysis Mediation Model, Independent Variables as Continuous Variables

## References

[1] Public Health England Patterns and trends in child obesity. Accessed December 10, 2018. https://app.box.com/s/og3q86aqejc99okxe9xyvpfvo21xai21/file/393885709361

[2] Commission on Ending Childhood Obesity Report of the commission on ending childhood obesity. Published 2016 Accessed December 10, 2018. https://apps.who.int/iris/bitstream/handle/10665/204176/9789241510066_eng.pdf;jsessionid=A8B7E1F743A314DDAFEEE69F6C20BC93?sequence=1

[3] World Health Organization Obesity and overweight. Published February 16, 2016 Accessed July 20, 2017. https://www.who.int/mediacentre/factsheets/fs311/en/

[4] Department of Health/Department for Children Schools and Families Sedentary behaviour and obesity: review of the current scientific evidence. 2010 Accessed December 10, 2018. https://assets.publishing.service.gov.uk/government/uploads/system/uploads/attachment_data/file/833151/dh_128225.pdf

[5] Tremblay MS, LeBlanc AG, Kho ME, Systematic review of sedentary behaviour and health indicators in school-aged children and youth. Int J Behav Nutr Phys Act. 2011;8:98. doi:10.1186/1479-5868-8-98 21936895 PMC3186735

[6] Vandewater EA, Shim MS, Caplovitz AG Linking obesity and activity level with children’s television and video game use. J Adolesc. 2004;27(1):71-85. doi:10.1016/j.adolescence.2003.10.003 15013261

[7] Weaver JB III, Mays D, Sargent Weaver S, Health-risk correlates of video-game playing among adults. Am J Prev Med. 2009;37(4):299-305. doi:10.1016/j.amepre.2009.06.014 19765501

[8] Marshall SJ, Biddle SJH, Gorely T, Cameron N, Murdey I Relationships between media use, body fatness and physical activity in children and youth: a meta-analysis. Int J Obes Relat Metab Disord. 2004;28(10):1238-1246. doi:10.1038/sj.ijo.0802706 15314635

[9] Desai RA, Krishnan-Sarin S, Cavallo D, Potenza MN Video-gaming among high school students: health correlates, gender differences, and problematic gaming. Pediatrics. 2010;126(6):e1414-e1424. doi:10.1542/peds.2009-2706 21078729 PMC3678538

[10] Kautiainen S, Koivusilta L, Lintonen T, Virtanen SM, Rimpelä A Use of information and communication technology and prevalence of overweight and obesity among adolescents. Int J Obes (Lond). 2005;29(8):925-933. doi:10.1038/sj.ijo.0802994 15925961

[11] Francis SL, Stancel MJ, Sernulka-George FD, Broffitt B, Levy SM, Janz KF Tracking of TV and video gaming during childhood: Iowa Bone Development Study. Int J Behav Nutr Phys Act. 2011;8(100). doi:10.1186/1479-5868-8-100 21943061 PMC3186734

[12] Hesketh K, Wake M, Graham M, Waters E Stability of television viewing and electronic game/computer use in a prospective cohort study of Australian children: relationship with body mass index. Int J Behav Nutr Phys Act. 2007;4(60). doi:10.1186/1479-5868-4-60 18021422 PMC2228322

[13] Dubois L, Farmer A, Girard M, Peterson K Regular sugar-sweetened beverage consumption between meals increases risk of overweight among preschool-aged children. J Am Diet Assoc. 2007;107(6):924-934. doi:10.1016/j.jada.2007.03.004 17524711

[14] Johnson RK Changing eating and physical activity patterns of US children. Proc Nutr Soc. 2000;59(2):295-301. doi:10.1017/S002966510000032X 10946798

[15] Malik VS, Willett WC, Hu FB Sugar-sweetened beverages and BMI in children and adolescents: reanalyses of a meta-analysis. Am J Clin Nutr. 2009;89(1):438-439. doi:10.3945/ajcn.2008.26980 19056589

[16] Chaput JP, Visby T, Nyby S, Video game playing increases food intake in adolescents: a randomized crossover study. Am J Clin Nutr. 2011;93(6):1196-1203. doi:10.3945/ajcn.110.008680 21490141

[17] Kenney EL, Gortmaker SL United States adolescents’ television, computer, videogame, smartphone, and tablet use: associations with sugary drinks, sleep, physical activity, and obesity. J Pediatr. 2017;182:144-149. doi:10.1016/j.jpeds.2016.11.015 27988020

[18] Wolfe J, Kar K, Perry A, Reynolds C, Gradisar M, Short MA Single night video-game use leads to sleep loss and attention deficits in older adolescents. J Adolesc. 2014;37(7):1003-1009. doi:10.1016/j.adolescence.2014.07.013 25118041

[19] García-Hermoso A, Ramírez-Vélez R, Saavedra JM Exercise, health outcomes, and pædiatric obesity: a systematic review of meta-analyses. J Sci Med Sport. 2019;22(1):76-84. doi:10.1016/j.jsams.2018.07.006 30054135

[20] Cappuccio FP, Taggart FM, Kandala NB, Meta-analysis of short sleep duration and obesity in children and adults. Sleep. 2008;31(5):619-626. doi:10.1093/sleep/31.5.619 18517032 PMC2398753

[21] Centre for Longitudinal Studies Millennium Cohort Study. Accessed April 4, 2019. https://cls.ucl.ac.uk/cls-studies/millennium-cohort-study/

[22] Centre for Longitudinal Studies Welcome to the Millennium Cohort Study. Accessed August 5, 2017. http://www.cls.ioe.ac.uk/page.aspx?&sitesectionid=851&sitesectiontitle=Welcome+to+the+Millennium+Cohort+Study

[23] Emerson E, Robertson J, Baines S, Hatton C Obesity in British children with and without intellectual disability: cohort study. BMC Public Health. 2016;16:644. doi:10.1186/s12889-016-3309-1 27460572 PMC4962444

[24] Laverty AA, Magee L, Monteiro CA, Saxena S, Millett C Sugar and artificially sweetened beverage consumption and adiposity changes: National longitudinal study. Int J Behav Nutr Phys Act. 2015;12:137. doi:10.1186/s12966-015-0297-y 26503493 PMC4624385

[25] Wright CM, Booth IW, Buckler JM, Growth reference charts for use in the United Kingdom. Arch Dis Child. 2002;86(1):11-14. doi:10.1136/adc.86.1.11 11806873 PMC1719041

[26] Anyaegbu G Using the OECD equivalence scale in taxes and benefits analysis. Econ Lab Market Rev. 2010;4:49–54.

[27] Klebanoff MA, Cole SR Use of multiple imputation in the epidemiologic literature. Am J Epidemiol. 2008;168(4):355-357. doi:10.1093/aje/kwn071 18591202 PMC2561989

[28] Mei Z, Grummer-Strawn LM Standard deviation of anthropometric Z-scores as a data quality assessment tool using the 2006 WHO growth standards: a cross country analysis. Bull World Health Organ. 2007;85(6):441-448. doi:10.2471/BLT.06.034421 17639241 PMC2636355

[29] Baron RM, Kenny DA The moderator-mediator variable distinction in social psychological research: conceptual, strategic, and statistical considerations. J Pers Soc Psychol. 1986;51(6):1173-1182. doi:10.1037/0022-3514.51.6.1173 3806354

[30] Streiner DL Finding our way: an introduction to path analysis. Can J Psychiatry. 2005;50(2):115-122. doi:10.1177/070674370505000207 15807228

[31] Klein RB Principles and Practice of Structural Equation Modeling. Guilford; 1998.

[32] Exelmans L, Van den Bulck J Sleep quality is negatively related to video gaming volume in adults. J Sleep Res. 2015;24(2):189-196. doi:10.1111/jsr.12255 25358428

[33] Brondel L, Romer MA, Nougues PM, Touyarou P, Davenne D Acute partial sleep deprivation increases food intake in healthy men. Am J Clin Nutr. 2010;91(6):1550-1559. doi:10.3945/ajcn.2009.28523 20357041

[34] Nedeltcheva AV, Kilkus JM, Imperial J, Kasza K, Schoeller DA, Penev PD Sleep curtailment is accompanied by increased intake of calories from snacks. Am J Clin Nutr. 2009;89(1):126-133. doi:10.3945/ajcn.2008.26574 19056602 PMC2615460

[35] Watanabe M, Kikuchi H, Tanaka K, Takahashi M Association of short sleep duration with weight gain and obesity at 1-year follow-up: a large-scale prospective study. Sleep. 2010;33(2):161-167. doi:10.1093/sleep/33.2.161 20175399 PMC2817903

[36] Lorenzon K, Russell C From apathy to ambivalence: how is persuasion knowledge reflected in consumers’ comments about in-game advertising? J Mark Commun. 2012;18(1):55-67. doi:10.1080/13527266.2011.620768

[37] Kresse C Brands in eSports – Red Bull: king of content marketing. Published March 14, 2016 Accessed May 12, 2017.http://esports-marketing-blog.com/red-bull-esports-marketing/#.WRW-zVXyu71.

[38] Yang M, Roskos-Ewoldsen DR, Dinu L, Arpan LM The effectiveness of “in-game” advertising: comparing college students’ explicit and implicit memory for brand names. J Advert. 2006;35(4):143-152. doi:10.2753/JOA0091-3367350410

[39] Anderson JR, Bower GH Human Associative Memory. Halstead; 1973.

[40] Reinehr T, Lass N, Toschke C, Rothermel J, Lanzinger S, Holl RW Which amount of BMI-SDS reduction is necessary to improve cardiovascular risk factors in overweight children? J Clin Endocrinol Metab. 2016;101(8):3171-3179. doi:10.1210/jc.2016-1885 27285295

[41] Marsh S, Foley LS, Wilks DC, Maddison R Family-based interventions for reducing sedentary time in youth: a systematic review of randomized controlled trials. Obes Rev. 2014;15(2):117-133. doi:10.1111/obr.12105 24102891

[42] Mallinckrodt V, Mizerski D The effects of playing an advergame on young children’s perceptions, preferences, and requests. J Advert. 2007;36(2):87-100. doi:10.2753/JOA0091-3367360206

[43] Hebert JR, Clemow L, Pbert L, Ockene IS, Ockene JK Social desirability bias in dietary self-report may compromise the validity of dietary intake measures. Int J Epidemiol. 1995;24(2):389-398. doi:10.1093/ije/24.2.389 7635601

[44] Bo S, De Carli L, Venco E, Impact of snacking pattern on overweight and obesity risk in a cohort of 11- to 13-year-old adolescents. J Pediatr Gastroenterol Nutr. 2014;59(4):465-471. doi:10.1097/MPG.0000000000000453 24897170

[45] Cleobury L, Tapper K Reasons for eating ‘unhealthy’ snacks in overweight and obese males and females. J Hum Nutr Diet. 2014;27(4):333-341. doi:10.1111/jhn.12169 24134077

