# Supplementary material for: Association of Video Game Use With Body Mass Index and Other Energy-Balance Behaviors in Children
Source: JAMA Pediatr. 2020 Apr 6;174(6):1–10. doi: 10.1001/jamapediatrics.2020.0202 (PMC7136857; doi:10.1001/jamapediatrics.2020.0202)
Supplement: Supplement. — eFigure 1. Exploratory Mediation Model (All Energy Balance Behaviours at Age 7) eFigure 2. Exploratory Mediation Model (Energy Balance Behaviours at Age 7 Minus Physical Activity) eFigure 3. Sensitivity Analysis Mediation Model, BMI-SDS Change From Age 5 to Age 14 as Outcome eFigure 4. Sensitivity Analysis Mediation Model, Independent Variables as Continuous Variables [file jamapediatr-174-563-s001.pdf]

Supplementary Online Content

Goodman W, Jackson SE, McFerran E, Purves R, Redpath I, Beeken RJ. Association of video game use with body mass index and other energy-balance behaviors in children. *JAMA Pediatr*. Published online April 6, 2020. doi:10.1001/jamapediatrics.2020.0202

eFigure 1. Exploratory Mediation Model (All Energy Balance Behaviours at Age 7)

eFigure 2. Exploratory Mediation Model (Energy Balance Behaviours at Age 7 Minus Physical Activity)

eFigure 3. Sensitivity Analysis Mediation Model, BMI-SDS Change From Age 5 to Age 14 as Outcome

eFigure 4. Sensitivity Analysis Mediation Model, Independent Variables as Continuous Variables

This supplementary material has been provided by the authors to give readers additional information about their work.

©2020 Goodman W et al. *JAMA Pediatrics*

eFigure 1. Exploratory Mediation Model (All Energy Balance Behaviours at Age 7)

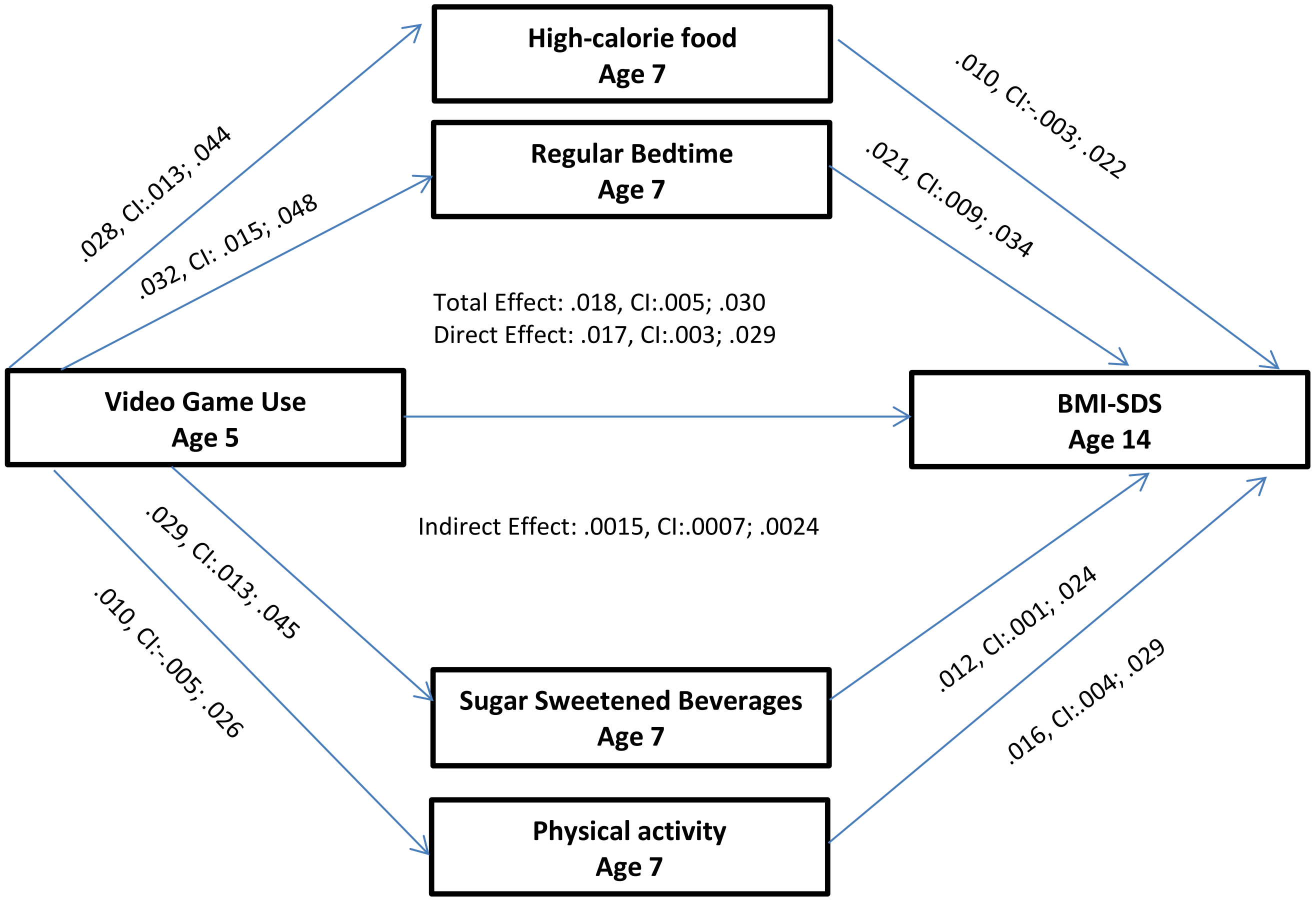

Model fit statistics: CFI= .989; TLI= .896; RMSEA= .038. Note: Standardized regression coefficients with the arrows pointing to the outcome variable.

©2020 Goodman W et al. *JAMA Pediatrics*

eFigure 2. Exploratory Mediation Model (Energy Balance Behaviours at Age 7 Minus Physical Activity)

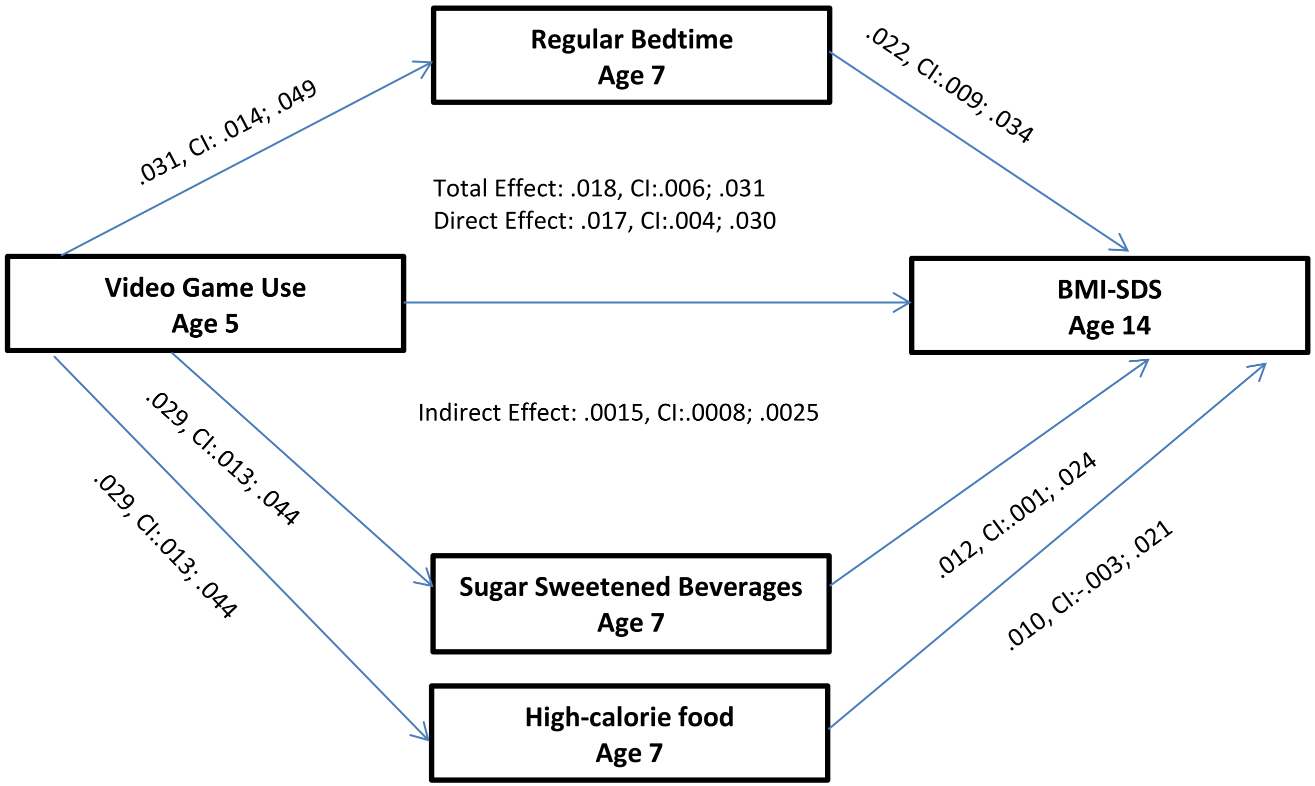

Model fit statistics: CFI= .989; TLI= .841; RMSEA= .047. Note: Standardized regression coefficients with the arrows pointing to the outcome variable.

©2020 Goodman W et al. *JAMA Pediatrics*

eFigure 3. Sensitivity Analysis Mediation Model, BMI-SDS Change From Age 5 to Age 14 as Outcome

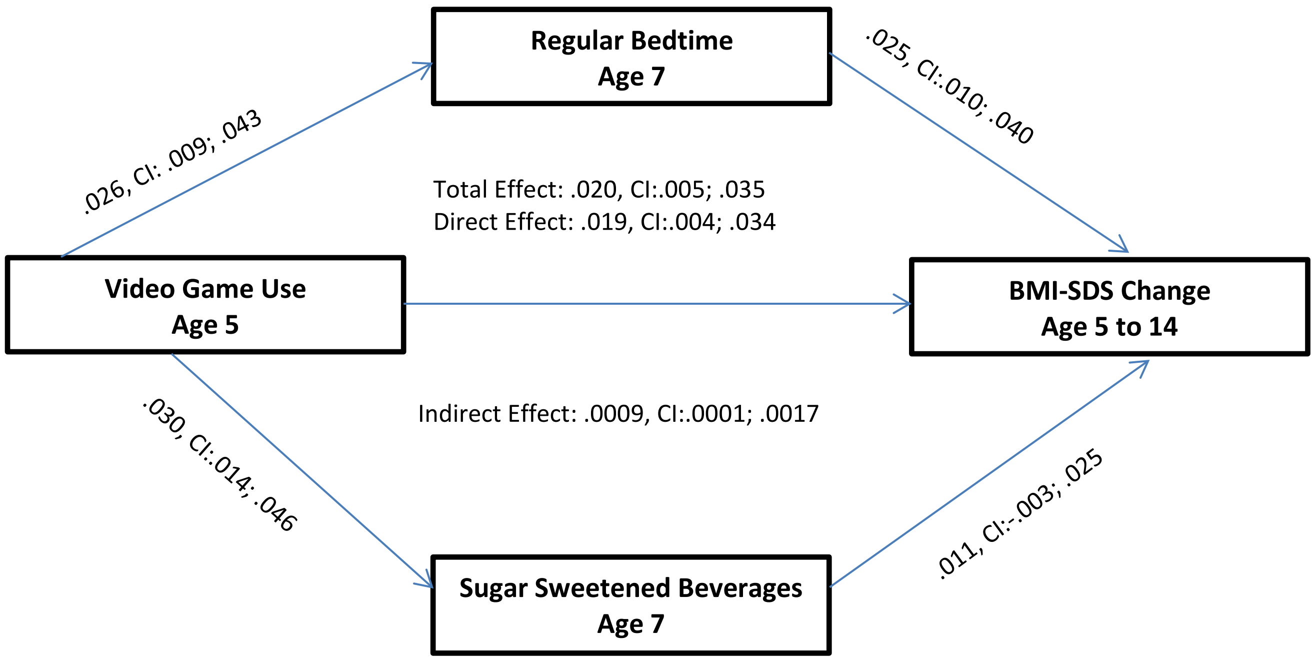

Model fit statistics: CFI= .999; TLI= .950; RMSEA= .021. Note: Standardized regression coefficients with the arrows pointing to the outcome variable.

©2020 Goodman W et al. *JAMA Pediatrics*

eFigure 4. Sensitivity Analysis Mediation Model, Independent Variables as Continuous Variables

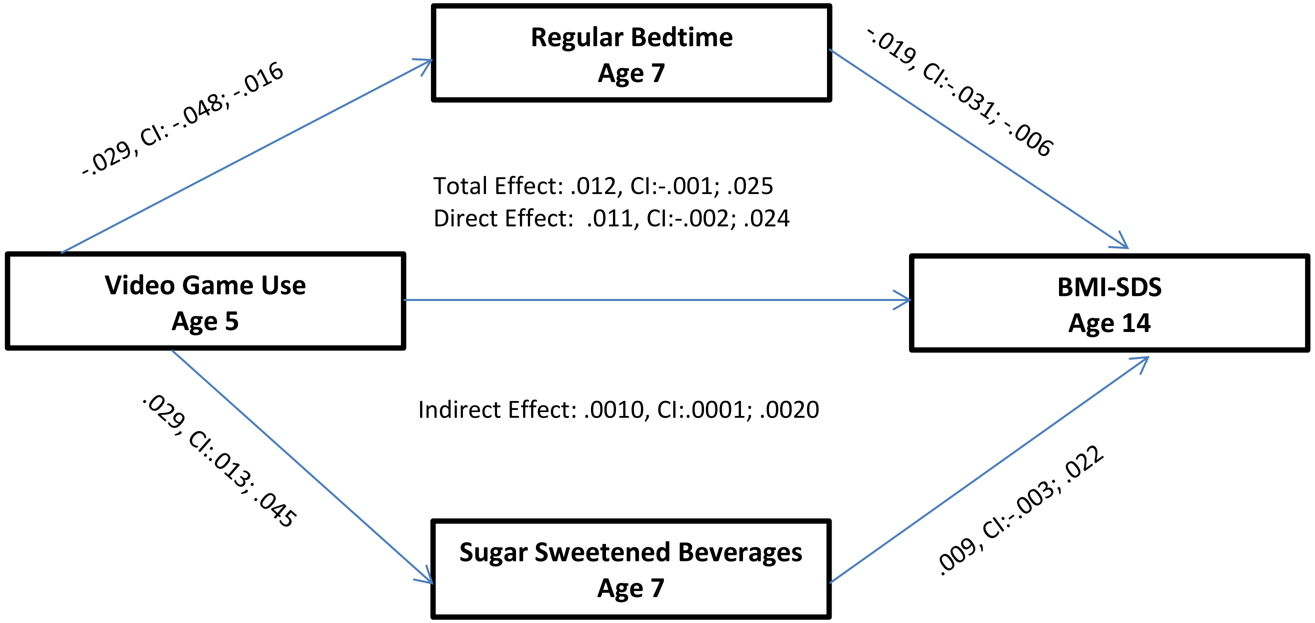

Model fit statistics: CFI= .999; TLI= .961; RMSEA= .026. Note: Standardized regression coefficients with the arrows pointing to the outcome variable; Regular Bedtime 1-Never regular, 2-Sometimes regular, 3-Usually regular, 4-Always regular; Video game use 1-None, 2-Less than an hour, 3-1 hour to less than 3 hours, 4-3 hours to less than 5 hours, 5-5 hours to less than 7 hours, 6-7 hours or more.

©2020 Goodman W et al. *JAMA Pediatrics*
